# Monthly Report of Diseases

**Published:** 1801-05

**Authors:** 


### MONTHLY REPORT of DISEASES,

Admitted under the Care of the PHYSICIANS of the FINSBURY DISPENSARY, St. John's Square, Clerkenwell.

From March 20, to April 20, 1801.

| | No. of Cafes. | | No. of Cafes. |
|---|---|---|---|
| Chlorofis and Amenorrhœa | 26 | Infantile Difeafes - - - - | 33 |
| Menorrhagia - - - - - | 4 | Anafarca - - - - - - | 3 |
| Diarrhœa - - - - - - | 11 | Cephalæa - - - - - - | 5 |
| Tuffis and Dyfpnœa - - | 39 | Epilepfy - - - - - - | 3 |
| Phthifis Pulmonalis - - | 9 | Hyfteria - - - - - - | 4 |
| Cynanche Tonfillarum - | 2 | Afthenia - - - - - - | 28 |
| Eryfipelas - - - - - - | 10 | Hypochondriafis and Dyfpepfia - - - - - - | 19 |
| Continued Fever - - - | 31 | | |
| Chronic Eruptions - - | 29 | | |

At this feafon of the year, fcrophulous affections are more particularly apt to fhew themfelves, in habits which have any tendency to the difeafe. A circumftance that may feem to oppofe an opinion that has arifen, and in fome meafure is fupported by the obfervation, that thefe complaints prevail more particularly in the northern regions of the world. It fhould, however, be confidered, that the winter's cold, which has been gradually operating, cannot have produced its *full* effect upon the conftitution until the commencement of the fpring. The *anti-vital* principles of cold cannot fail to produce debility, and all its numerous offspring of difeafes.

In the more northern parts of this ifland, few families, comparatively, are altogether free from fcrophulous contamination. Even in this metropolis, inftances not unfrequently are found in which the difeafe appears, accompanied with all its dangerous and difagreeable fymptoms.

A confiderable number of thefe has fallen within the diftrict of the Finfbury Inftitution.

Sometimes this complaint fhews itfelf in inflammation and ulceration on the edges of the eye-lids, which is apt to occafion an entire, or partial, lofs of the lafhes, and in confequence of being communicated to the eye itfelf, frequently renders the exercife of that organ, not only painful and inconvenient to the patient, but fometimes, by inducing blindnefs, deprives him entirely of its ufe.

Scrophula, in many cafes, affumes the form of phthifis, which indeed may in general be regarded as an *internal* fcrophula.

Frequently this complaint fhews its malignant efficacy in producing ulcers and indolent tumours in various parts of the body, leaving indelible traces, disfiguring the form, impairing the the eafy and vigorous motions of the limbs, and inducing a variety of difeafes, in which, if not altogether incurable, the patient has feldom any thing to hope but from fubmitting himfelf to the certain pain, and rifking the precarious refult of a furgical operation.

By far the moft frequent fhape in which the difeafe has fubjected itfelf to the obfervation of the Reporter, is an obftruction in the mefenteric glands. All the perfons affected with the tabes mefenterica were young children.

Of the cafes of fcrophula, indeed, few have occurred amongft adults; a circumftance which, in the clafs of the extremely poor in London, may in part arife from their too feldom being able to provide for their puny and difeafed offspring, even a fcanty and occafional fupply of that nourifhing and ftrengthening diet, peculiarly requifite for the fupport of a fcrophulous conftitution.

In the treatment of fcrophula, the writer of this article prefcribed cleanlinefs, exercife, cold bathing, and as much of fubftantial food as the circumftances of the patient, or thofe of his family, would enable him conveniently to procure.

As one of the beft correctors of a relaxed and debilitated habit, port wine was in fome inftances ftrongly recommended; but the expence of this article rendering it at prefent almoft inacceffible to the greater part of Difpenfary-patients, it was in general found neceffary to fubftitute the Peruvian or fome other of the barks that are made ufe of in medicine.

The multitude of general remedies that have been propofed for the treatment of fcrophula, demonftrate the difficulty of accomplifhing a cure. Each has, in its turn, been at one time warmly applauded, and eagerly received; at another, as bitterly reprobated, and as generally rejected. Millepedes and burnt fponge, antimony and mercury, faffafras and mezereum, tuffilago and cicuta, have fucceffively had their career of triumph, and their days of difgrace; nor is it too bold to prognofticate, that the period will arrive when they will fhare the fate of many other remedies which have now funk into neglect, and which repofe in the fame peaceful oblivion with the afhes of their authors. Tonic remedies, indeed, have inherited, and feem likely to enjoy, a more permanent reputation. Inftead of requiring correction, Nature, in the difeafe at prefent under confideration, feems to be more in need of our friendly fupport and affiftance. The internal remedies, from which the greateft fuccefs may be expected, are, the Peruvian bark, already mentioned, with the various preparations of fteel, in conjunction, perhaps, with gently ftimulating aperients, the muriated tincture of iron, in dofes of from five to ten drops, poured from from a two-ounce vial, three times a day, has been given with ſignal advantage in a variety of ſcrophulous affections.

The barytic muriate has of late been propoſed by Dr. Crawford, and appears, from the teſtimonies of many reſpectable authorities, to be well deſerving of further attention. But it is neceſſary to caution the practitioner, who may be induced to give it a trial, againſt the admixture of noxious metals, with which the barytic ſolution is ſometimes contaminated. Its impurity may be always detected by the addition of a ſmall quantity of barytic lime-water. The doſe ſhould be carefully regulated, and gradually increaſed, leſt it produce ſymptoms of nervous affection. It may be uſeful to remark, that twenty drops at a time is as much as an adult can bear with impunity.

The remedy which ſeemed to have the moſt ſtriking, and the only one perhaps that had a permanent effect in alleviating the ſymptoms, or in abridging the period of the diſorder, was a temporary reſidence at the ſea-ſide. This afforded an opportunity to the patient of experiencing at once the ſalubrious influence of the elements, both of which have been found eminently conducive to the eaſe or the relief of ſcrophulas, as well as all diſeaſes which have their ſource in phyſical debility.

One of the moſt decided and obſtinate caſes of ſcrophulous affections, which was alluded to in one of the preceding Reports, in which the patient ſuffered alternately from a ſore in her breaſt, and a violent pain in her head; her complaint had been of long ſtanding, and had not in the ſlighteſt degree yielded to the remedies which had been adminiſtered to her before her application for relief to the Finſbury Diſpenſary. She ſeemed, for a time, to be relieved by the Cort. Peruv. taken regularly in frequent and conſiderable doſes.

After a trial of ſome weeks the caſe continued to be imperfect. It was thought right to recommend, as the only chance that remained of a perfect reſtoration to her former health and vigour, to ſettle, during a conſiderable period of the ſummer, on the ſea-coaſt. In conſequence of a faithful obedience to this advice, her conſtitution ſeems ſince to be in a great meaſure regenerated, and the affections, which before were ſo troubleſome to her, entirely removed.

The preceding obſervations, with regard to the ſalutary efficacy of ſea-bathing, ought to be qualified by the exception of its uſe, in thoſe inſtances where there is any morbid affection of the lungs.

In theſe caſes, the ſea generally aggravates the ſufferings of the patient, and accelerates the termination of his life.

How large a number of the deaths we ſee inſerted in the public papers is that of thoſe which have occurred at ſea-bathinging-places, where it is generally obſerved, that the deceaſed had gone for the benefit of his health.

The more than ordinary expedition with which Death executes his deſtined taſk in theſe faſhionable reſorts of gaiety and ſickneſs, is ſtrikingly exhibited to our view in their crowded records of mortality.

The authentic and melancholy enumeration of the victims to untimely fate, one ſhould imagine, could ſcarcely fail to awaken a gloomy preſentiment in the mind, that in ſome meaſure tends to counteract the diſpoſition to hope, ſo eaſily imbibed and ſo anxiouſly cheriſhed by the multitude of conſumptive invalids, who, on the approach of each returning ſummer, haſten with eagerneſs to the coaſt, fondly expecting to find, amidſt the breezes of the ocean, that relief which, elſewhere, had been ſought for in vain.

*Red Lion Square.* J. R.

